# Mechanisms of trastuzumab induced cardiotoxicity – is exercise a potential treatment?

**DOI:** 10.1186/s40959-023-00172-3

**Published:** 2023-04-25

**Authors:** Holden Eaton, Kerstin Nina Timm

**Affiliations:** 1 Merton College, University of Oxford, Merton St, Oxford, OX1 4JD UK; 2 Department of Pharmacology, University of Oxford, Mansfield Road, Oxford, OX1 3QT UK

**Keywords:** Trastuzumab, Cardiotoxicity, Exercise, Metabolism, Chemotherapy, Heart failure, Cardioprotection

## Abstract

The use of the adjuvant therapeutic antibody trastuzumab in breast cancer is associated with a range of cardiotoxic side effects despite successfully reducing the severity of outcomes cancer patients,. The most common cardiac effect, a reduction in left ventricular ejection fraction (LVEF), is a known precursor to heart failure and often requires interruption of chemotherapy to avoid endangering patients further. An understanding of trastuzumab’s cardiac-specific interactions is therefore critical in devising new methods to not only avoid permanent cardiac damage, but also prolong treatment time, and therefore effectiveness, for breast cancer patients. Increasingly, the use of exercise as a treatment has been indicated across the field of cardio-oncology due to encouraging evidence that it can protect against LVEF reductions and heart failure. This review explores the mechanisms of trastuzumab-mediated cardiotoxicity, as well as the physiological effects of exercise on the heart, in order to assess the suitability of exercise intervention for breast cancer patients on trastuzumab antibody-therapy. We furthermore draw comparison to existing evidence for exercise intervention as a cardioprotective treatment in doxorubicin-induced cardiotoxicity. Although preclinical evidence seems to support exercise-based approaches also in trastuzumab-cardiotoxicity, current clinical evidence is too limited to confidently recommend it as a treatment, largely owing to issues of adherence. Future studies should therefore examine how the variety and duration of exercise can be adjusted to improve treatment effectiveness at a more personalised level.

## Introduction

Trastuzumab is a recombinant humanized monoclonal antibody selective for the extracellular domain of the human epidermal growth factor receptor protein (HER2), whose overexpression in 20–30% of breast cancers (BC) [1] is associated with poor prognosis [2]. Having been approved for medical use in 1998 in the US, trastuzumab was among the first successful targeted therapies, greatly improving treatment outcomes when used in combination with traditional chemotherapy such as the anthracycline doxorubicin. Phase III clinical trials showed an increased time to disease progression, longer response duration and increased survival times when trastuzumab was added to standard chemotherapy during treatment of metastatic BC [3]. However, despite its clear success in the treatment of malignancy, trastuzumab’s mechanism of action also lends itself to some significant cardiotoxic side-effects. HER2 receptors are not limited to tumour cells, but are in fact highly expressed in cardiomyocytes, likely participating in a variety of cardioprotective mechanisms in response to cell stressors [4]. As a result, the use of trastuzumab can lead to significantly decreased left-ventricular ejection fraction (LVEF) [5] and ultimately heart failure (HF) [6–8] when administered alone, and can amplify the effects of other known cardiotoxic chemotherapies (e.g. anthracyclines) when used in combination [3, 6, 9]. Alongside potentially irreversible cardiovascular decline [8, 10, 11], treatment-induced cardiotoxicity (TIC) may require interruption of therapy, which itself can have potentially serious repercussions with respect to overall patient survival [12]. As such, development of treatment strategies to limit the cardiotoxicity of trastuzumab adjuvant therapy is critically important to improve its success.

Exercise is known to modulate cardiomyocyte behaviour, improving both metabolic and contractile function when undertaken regularly. Observations that higher levels of physical activity and chronic exercise are associated with reduced incidence [13–15] and even reversal of the pathological remodelling of HF [16, 17] have prompted suggestions that exercise could be used to help prevent or reverse the cardiotoxicity of trastuzumab. In this review we evaluate the evidence for exercise as a treatment, through exploring both the mechanisms of trastuzumab-mediated cardiotoxicity and the counteracting actions of exercise adaptation, and comparing this with findings from clinical studies and evidence from doxorubicin-induced cardiotoxicity.

## Trastuzumab-mediated cardiotoxicity in patients

In 1998 trastuzumab was classed separately to other known cardiotoxic cancer treatments, such as anthracyclines, under the cancer therapeutics-related cardiac dysfunction (CTRCD) classifications [18]. As a CTRCD type-II agent, trastuzumab was thought to be less concerning than its type-I counterparts (anthracyclines), considering its dose-independent cardiotoxicity and apparent lack of cardiomyocyte cell death following its use [19]. Any toxic effects owing to trastuzumab were generally considered to be reversible following cessation of treatment, while anthracycline-mediated cardiac damage can persist despite treatment cessation [20]. However, recent evidence has indicated trastuzumab-related cardiotoxicity should, in fact, be more of a clinical concern.

Various clinical trials and meta-analyses have reported significant incidences of cardiac dysfunction (CD), including both LVEF decline and congestive HF, among patients receiving trastuzumab (Table 1). Other studies indicate that this CD may be disruptive enough to require treatment interruption or cessation. Reviews of multiple randomised controlled trials (RCTs), observational studies and major trastuzumab adjuvant trials indicate that up to 43% of women treated with trastuzumab will develop some form of CD requiring treatment discontinuation [8, 21, 22]. Despite minimising the cardiovascular impact of trastuzumab, treatment interruption can be detrimental to the overall patient health through potentially increasing risk of cancer recurrence. In one study, patients for whom trastuzumab treatment had been interrupted displayed 3-fold decreased rates of disease-free and overall survival which were likely related to cancer recurrence [12]. 62% of these patients reported treatment-induced cardiotoxicity (TIC) as the reason for treatment interruption.

**Table 1:** Trastuzumab-mediated cardiotoxicity rates in patients across various studies

Study	Observation
*Seidman et al.* [23]	**Cardiovascular decline**, defined as decreased LVEF or symptoms of congestive heart failure (CHF), present in **7% of patients** when **trastuzumab** was used as a single agent
*Calvillo-Arguelles et al.* [21]	3-year cumulative incidence of **cardiotoxicity** was **35%** (defined as a ≥ 10% decline in LVEF, to < 55% without symptoms OR ≥ 5% decrease in LVEF, to < 55% with symptoms, in at least 1 MUGA scan) for women diagnosed with **HER2 positive breast cancer**, and who had **received > 12 months of trastuzumab therapy**
*Chen et al.* [7]	Meta-analysis of 10 randomised controlled trials (RCT) where incidence of **CHF** and **LVEF decrease** were **1.9%** and **7.5%** respectively in **HER2 positive breast cancer patients receiving trastuzumab**
*Thavendiranathan et al.* [24]	Cumulative incidence of hospitalisation/emergency room visits for CHF, outpatient diagnosis of **CHF** or **cardiovascular death** was **5.1%** for **trastuzumab treatment without anthracyclines**

In addition to its acute effects which may require treatment interruption, trastuzumab-induced cardiotoxicity can also be irreversible in some cases, contradicting initial assumptions about the drug’s long-term effects. A meta-analysis shows that during follow-up cardiac evaluation of patients who had received trastuzumab treatment as an adjuvant to either anthracyclins, paclitaxel, docetaxel or carboplatin up to 4% showed severe cardiotoxic side-effects [8]. However, since Trastuzumab was given in combination with chemotherapy the direct effect of trastuzumab on cardiac function in isolation is difficult to estimate. Furthermore, a prospective observational study of patients receiving trastuzumab treatment revealed 6/38 TIC cases were irreversible despite optimal cardioprotective therapy [10]; while another study showed lower mean LVEF in BC patients with history of trastuzumab-related cardiotoxicity, than in patients without such history, even up to 7-years post-treatment [11]. Again, the latter study was performed in patients of whom 90% had received anthracycline chemotherapy prior to Trastuzumab, which precludes attribution of cardiac effects to trastuzumab alone. Furthermore, while cardiotoxicity is often defined as a decrease in LVEF of > 10% or to < 50%, this ignores the ever more prevalent diagnosis of heart failure with preserved ejection fraction (HFpEF), particularly in women. The current definition and screening criteria for HF in cancer patients are thus limited and should be expanded to include evaluating the risk of developing HFpEF.

These observations are of greater significance considering trastuzumab is often given to patients as an adjuvant to anthracycline treatment, and therefore its cardiotoxic effects can be amplified. An observational study of 12,500 women diagnosed with invasive BC over an 8-year period highlighted that only 3.5% received a combined treatment, but that these patients displayed the highest incidence of cardiomyopathy (Table 2) [6]. The same study also revealed that trastuzumab-only treatment resulted in a greater incidence of cardiomyopathy than anthracycline-only treatment (Table 2). Similar observations were echoed by *Thavendiranathan et al.* [24] – the estimated cumulative incidence of major cardiac events was greater for trastuzumab-only treatment as compared with anthracycline-only treatment – indicating that trastuzumab may even be more concerning than anthracycline when considering TIC.

**Table 2:** Cardiotoxicity rates in response to different treatments. (adapted from Bowles et al. [6])

	Treatment	Adjusted hazard ratio (95% CI) for Heart Failure or Cardiomyopathy
All ages (n = 12,500 women)	No chemotherapy	1.00
	Anthracycline only	1.40 (1.11 to 1.76)
	Trastuzumab only	4.12 (2.30 to 7.42)
	Anthracycline + trastuzumab	7.19 (5.00 to 10.35)

Therefore, interventions which might reduce trastuzumab-mediated cardiac dysfunction could be of benefit to HER2-positive BC patients who are at risk of developing detrimental cardiomyopathy from trastuzumab treatment.

## Mechanisms of trastuzumab-induced cardiotoxicity

**Fig. 1:**
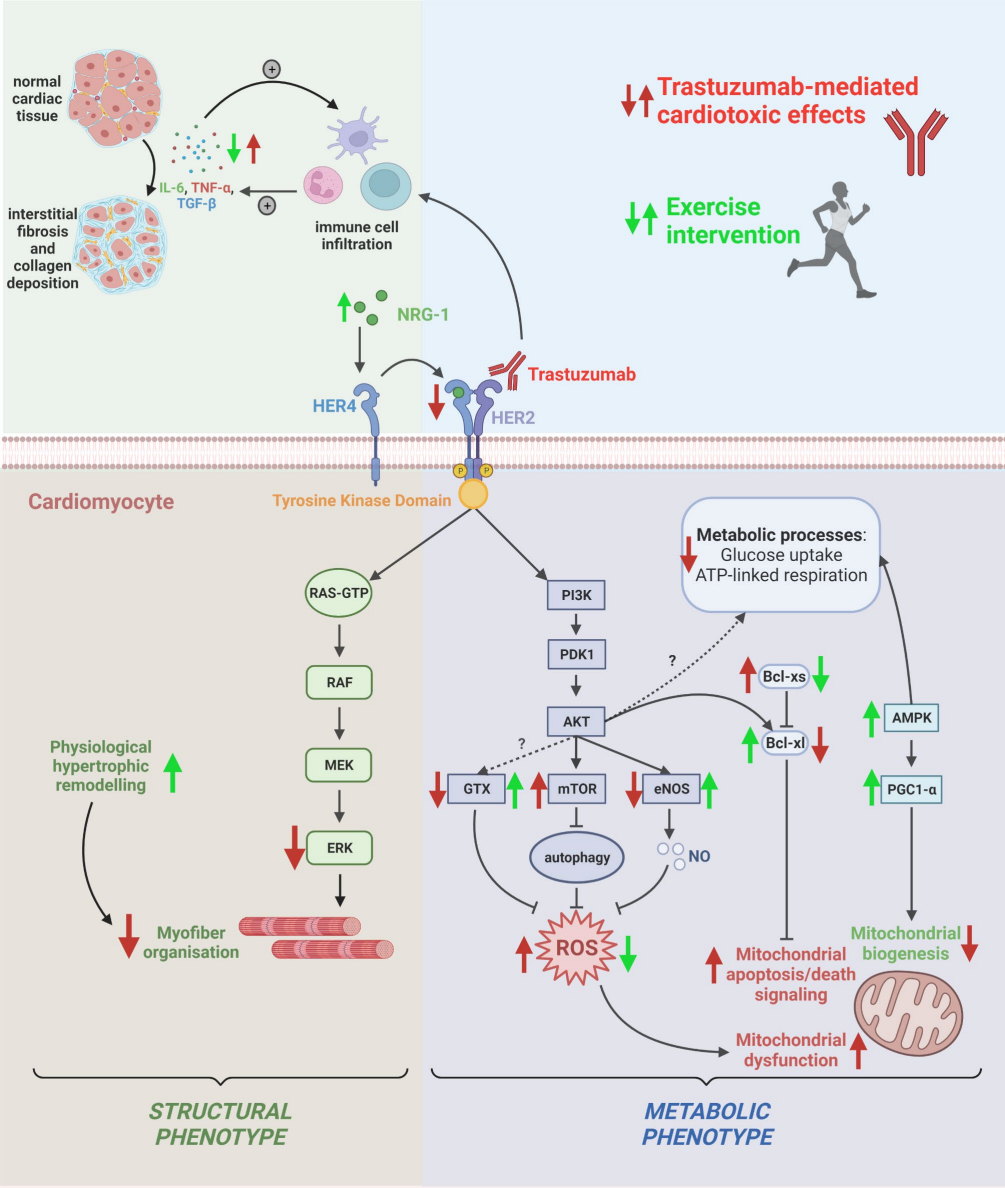
Summary of trastuzumab’s effects in cardiomyocytes (red arrows) and counteracting effects of exercise (green arrows) from human and pre-clinical animal studies

Before considering how exercise might be an appropriate intervention in trastuzumab-induced cardiotoxicity, it is critical to understand the molecular mechanisms which underly the pathological changes resulting from trastuzumab-mediated TIC. Trastuzumab binds an extracellular segment of the HER2 protein (Erbb2 in rodents), present on both tumour cells and cardiomyocytes. By doing so, it prevents heterodimerization of nearby HER3/4 receptors in response to neuregulin (NRG-1) signals, thereby inhibiting any further downstream signalling. Cardiac NRG1 signalling pathways via HER2 are protective, being upregulated in response to increased cardiovascular stress, such as exposure to other cardiotoxic agents like doxorubicin [25], and during development [26]. Protective pathways (Fig. 1) involve secondary messengers such as RAS/RAF, which upregulates ERK1/2 to stabilise myofibril structure and inhibit apoptosis, and PI3K/AKT, which increase mitochondrial respiration, eNOS activity [27] and reactive oxygen species (ROS) production. Conditional cardiac-restricted HER2 deletion in mice resulted in chamber dilation, wall thinning and decreased contractility – markers of dilated cardiomyopathy (DCM) [26] – demonstrating the importance of these protective pathways. Observations in both human foetal cardiomyocytes and rat cardiomyoblasts have shown that, through inhibiting normal NRG-1 signalling via HER2 receptors, trastuzumab disrupts downstream ERK1/2 and AKT activation [28, 29]. This triggers various pathological changes, both energetic and structural, which may ultimately lead to HF.

### Trastuzumab leads to oxidative stress and autophagy

Amongst the cardioprotective mechanisms impaired by trastuzumab-mediated HER2 inhibition are those regulating intracellular ROS – the factor responsible for oxidative stress. Mouse models of trastuzumab-toxicity show significant elevation of oxidative and nitrative damage by-products (NT and 4-HNE) in cardiomyocytes from treated animals [30]. This increase may be partially related to a reduction in ROS scavenging ability following decreased NRG-1/HER2 axis activation. Expression of glutathione, a known antioxidant enzyme, increases in ErbB2-overexpressing mice hearts, thereby contributing to significantly reduced ROS levels [31]. Similarly, NRG-1 signalling induced an increase in NO production (known ROS scavenger) through time-dependent phosphorylation of eNOS in neonatal rat cardiomyocytes [27]. Therefore, treatment-induced HER2 inhibition could reduce availability of antioxidant molecules like glutathione and NO, thereby increasing ROS levels. Indeed, mouse cardiac tissue catalase and glutathione peroxidase levels significantly decreased following trastuzumab treatment, which corresponded with an increase in tissue malondialdehyde (MDA – a marker of oxidative stress) [32].

However, findings in human models implicate alternative mechanisms in the process of ROS accumulation, highlighting the limitations of relying on pre-clinical animal models. It seems trastuzumab may also phosphorylate HER1/2 subunits, consequently upregulating mTOR signalling in human primary cardiomyocytes [33]. This was observed to downregulate autophagy, the intracellular ‘waste removal’ process, though inhibition of autophagosome formation proteins (e.g. Atg5-12/14, Beclin-1). The resulting accumulation of dysfunctional or damaged mitochondria and free radicals contribute to raised ROS production. Treatment of human induced pluripotent stem cell-derived cardiomyocytes (iPSC-CMs) with trastuzumab also led to dysfunctional mitochondria displaying altered energy metabolism pathways, as assessed by RNAseq analysis [34].

Cardiomyocytes exposed to trastuzumab display a diminished ability to adapt to increased oxidative stress from other sources, and are therefore more susceptible to the effects of other cardiotoxic agents. For example, anthracycline-mediated cardiotoxicity is partly the result of increased myocardial ROS generation via multiple mechanisms [35, 36], which is therefore exacerbated by trastuzumab-mediated reductions in antioxidant availability. This helps explain observations that adjuvant trastuzumab treatment following prior anthracycline-based therapy result in more severe cardiovascular decline [9], and a higher incidence of TIC [6, 3] .

## Trastuzumab impairs mitochondrial function and thus cardiac energetics

**Fig. 2:**
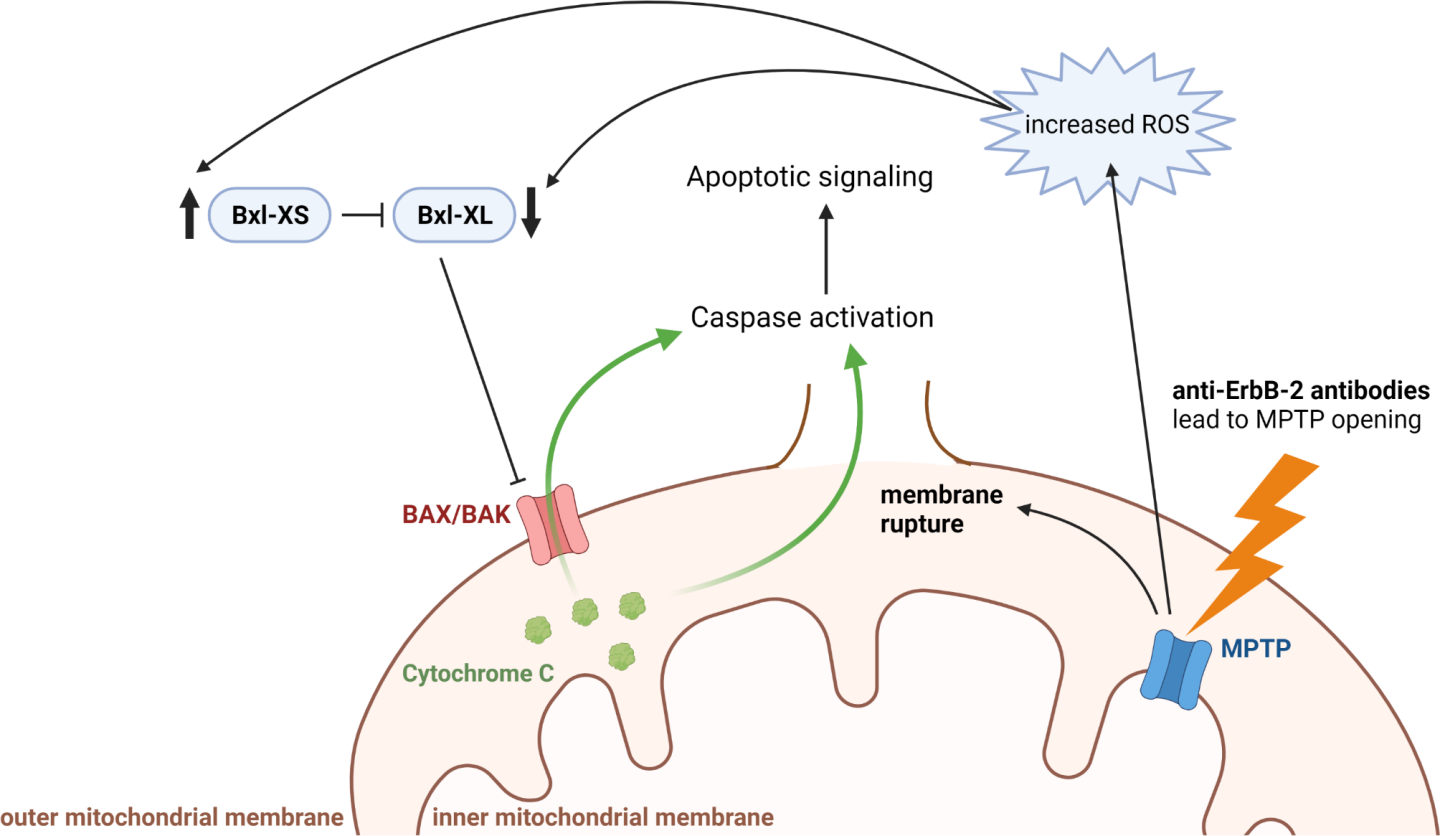
Trastuzumab treatment leads to apoptotic signalling in cardiomyocytes. The anti-ErbB2 antibody trastuzumab causes mitochondrial permeability transition pore (MPTP) opening and increased reactive oxygen species (ROS) production, both contributing to impaired mitochondrial function [30]. Cytochrome-c release from the mitochondrial intermembrane space promotes caspase activation and apoptotic signalling via BAX/BAK

Increased ROS can have various effects on cardiomyocyte function through altered physiological signalling and non-specific hyperoxidation of proteins. Clinical findings support a role for ROS in HF, as levels of pericardial [37] and serum [38] oxidative stress markers are correlated with worsening disease progression. Perhaps the most significant effect of increased ROS production, however, is declining mitochondrial function leading to metabolic impairment. Mitochondria are particularly sensitive to sudden increases in intracellular ROS levels, responding through a complex array of mechanisms including a loss of mitochondrial membrane integrity and release of cytochrome C (Fig. 2). Treatment of neonatal rat cardiomyocytes with ErbB2-directed antibodies led to raised ROS, followed by increased cytochrome C release, loss of mitochondrial membrane potential [29] and a shift in ratio of pro-/anti-apoptotic Bcl proteins leading to significant mitochondrial dysfunction [39] – although both papers used alternative anti-ErbB2 antibodies rather than trastuzumab. Similarly, ultrastructural analysis of rabbit myocytes following four doses of trastuzumab revealed morphological disruption of mitochondria indicative of membrane disruption [40]. As such, mitochondrial density within cardiomyocytes is reduced in mice treated with trastuzumab [30] and a corresponding 35% lower ATP output is observed in anti-Erbb2 antibody-treated rat ventricular myocytes [39]. Such metabolic disruption could be a significant contributor to the reduced cardiac output (CO) observed during TIC.

Once again, findings in human models of trastuzumab cardiotoxicity differ slightly – perhaps owing to the inconsistent use of anti-ErbB2 antibodies (other than trastuzumab) in some animal models. Evidence from human iPSC-CMs has shown that trastuzumab treatment may precipitate dysregulation of key cardiac energy metabolism genes and proteins, and that this may be a more prevalent contributor to metabolic dysfunction. Significantly reduced rates of ATP-linked respiration, glucose uptake [22] and mitochondrial content in treated human iPSC-CMs were correlated with altered phosphorylation states of key metabolic proteins, including AKT, mTOR and AMPKα1/2, as well as dysregulation of critical genes such as PGC1a^3^ [3]. Further gene ontology analyses also revealed downregulation of mitochondrial-associated genes involved with small molecule metabolism [22]. Therefore clearly there is a need to resolve the contrasting findings between animal and human preclinical models to ascertain which of these energetic mechanisms is occurring in patients.

### Trastuzumab leads to structural remodelling of the heart

Irregular cardiac structure is also a feature of preclinical models of trastuzumab-mediated TIC, although the findings are somewhat conflicting and clinical data haven not yet provided clarification. Given trastuzumab is an antibody, it may encourage pro-inflammatory effects in the heart triggering fibrosis and pathogenic tissue remodelling. Trastuzumab-treated mice displayed upregulated pro-inflammatory signalling, including overexpression of TNF-alpha [41], IL-6 and TGF-beta [32]. This was associated with increased diffuse infiltration of immune/inflammatory cells in cardiac tissues and significantly increased interstitial fibrosis. These changes are consistent with the pathological remodelling described in cardiac hypertrophy, however there is currently no evidence for a hypertrophic phenotype in clinical studies of trastuzumab cardiotoxicity.

In contrast, cardiac myofibers in trastuzumab-treated rats had a thinner, more stretched appearance [30], alongside decreased mitochondrial density – changes reflective of a phenotype like DCM. Similarly left-ventricular posterior wall thickness reduced by 17% in mice following a 7-day treatment period, and left ventricular systolic diameter increased by 19%^29^. As a potential underlying mechanism, ERK1/2 inhibition has been shown to cause myofibrillar disarray in rat cardiomyocytes, with similar effects also resulting from anti-ErbB2 antibody treatment [42]. However once again there is a lack of clear clinical evidence for DCM in patients treated with trastuzumab, although reductions in LVEF such as those described by *Chen et al.* [7] would be consistent with such a phenotype (as declining LVEF is a common marker of DCM).

## The role of exercise in cardiac function

Exercise represents an evolutionarily important short-term stressor which, through repeated exposure, triggers various physiological adaptations to improve cardiovascular performance. Adaptations in the heart are both metabolic and structural, improving resting and active cardiac output (CO). These changes are initiated by chemical and physical signals acting on signalling proteins and triggering cardioprotective transcription programmes. For individuals with cardiovascular pathology such as HF, regular exercise may therefore improve baseline cardiovascular markers. An RCT of regular exercise in patients with coronary artery disease revealed significantly increased baseline LVEF (> 30%) over 12 weeks [16]. Similar effects have been observed in patients with stable chronic HF [17]. Therefore we hypothesise that many of the cardioprotective changes triggered by exercise could help to reverse or counteract trastuzumab-mediated TIC.

### Pre-clinical evidence supports exercise as an intervention in trastuzumab-induced cardiotoxicity

Pre-clinical exercise models have highlighted multiple cardioprotective mechanisms relevant to the context of trastuzumab cardiotoxicity, although definitive evidence from trastuzumab-specific models is lacking. Exercise-trained rats display upregulated NRG-1 expression and ErbB2/PI3K/Akt signalling axis activity compared to inactive controls [43], indicating exercise-induced NRG-1 signalling could act to competitively disrupt trastuzumab-mediated HER2 inhibition. Furthermore, multiple rodent models have illustrated training-induced reductions in doxorubicin-mediated intracellular ROS increases. This includes significantly increased cardiac levels of glutathione [44], altered Bax-Bcl ratios [45] and decreased MDA levels [46]. Although not taken from a trastuzumab-specific model, this finding could have translational value given the interactions between trastuzumab and glutathione, as well as Bax/Bcl proteins.

Exercise training may also have an impact on cardiomyocyte metabolism. Increased activity of AMPK is a notable feature of cardiac adaptation to exercise in rats [47], while pharmacological activation of AMPK in trastuzumab-treated human iPSC-CMs increased glucose uptake and restoredmitochondrial respiratory capacity and contractile dysfunction [34]. Therefore by increasing AMPK activity, exercise could undo the metabolic impairment of trastuzumab TIC which may be sufficient to reverse the contractile dysfunction. In addition, there is clear evidence that aerobic exercise in rodents increases cardiac PGC-1a protein content – a crucial regulator of mitochondrial biogenesis, which is disrupted in trastuzumab-treated human iPSC-CMs [34]. Rats undergoing an intensive exercise regime displayed 44% higher cardiac PGC-1a protein content, alongside a 37% increase in mitochondrial density [48]. Similar effects were also observed in mouse exercise models [49], alongside increased mitochondrial capacity, fatty acid oxidation and glycogen synthesis [50].

Finally, there is also evidence that moderate exercise could offset the pro-inflammatory effects of trastuzumab and could therefore counteract any adverse remodelling. In a rat model of myocardial infarction-induced HF, aerobic exercise reduced plasma levels of TNF-alpha and IL-6 [51]. In addition, exercise (through repetitive volume-overloading of the heart) is known to counteract myofiber disorganisation through hypertrophic remodelling [52].

### Patient-based data is currently insufficient to confidently recommend exercise treatment

Clinical evidence in support of these findings is sparse, however, with only two patient studies exploring the effects of exercise specifically in the context of trastuzumab treatment. In their clinical trial, *Hojan et al.* describe a statistically significant decrease in LVEF in control patients, which was not observed in patients undergoing exercise treatment [53]. The exercise intervention occurred mid-treatment (9 week duration between 3 and 6 months of treatment) and consisted of both aerobic and resistance training. Other outcomes were evaluated, such as the 6 min walk test and serum biomarkers, but none of these showed significant differences. In addition, evidence from similar trials in doxorubicin cohorts is positive (Table 3), including the well-designed and rigorous BReast Cancer Randomized EXercise InTervention (BREXIT) trial [54], which provides evidence that continuing exercise post-treatment can be beneficial (rehab). These encouraging findings perhaps predict successes in trastuzumab patients given some of the similarities in cardiotoxicity mechanisms (e.g. ROS increases and metabolic disturbance).

**Table 3:** – Summary of clinical findings in trials examining exercise intervention for doxorubicin-mediated cardiotoxicity. Positive findings may predict similar success in trastuzumab-mediated cardiotoxicity given some mechanistic overlap between the two drugs

Reference	Patient Group	Intervention	Finding
*Kirkham et al., 2018* [62]	24 patients received (intervention group = 13) 60 mg/m^2^ of Dox and 600 mg/m^2^ of cyclophosphamide	24-48 h before each patient’s **first** doxorubicin treatment Supervised treadmill bout involving 10-min warm-up, 30 min (70% age-predicted HRR), 5 min cooldown	No difference in longitudinal strain, twist or cardiac troponin Mitigated increase in cardiac output, resting heart rate and decreased systemic vascular resistance relative to control group
*Lee et al., 2019* [63]	30 participants (intervention group = 15) following completion of 8-week anthracycline-based chemotherapy (every 2-weeks for 4 cycles)	3x weekly for 8 weeks HIIT sessions included 7 × (1-min interval 90% peak power output (PPO) followed by 2 min interval at 10% PPO)	VO2-max maintained in HIIT groups compared with significant decrease in control group
*Mijwel et al., 2018* [64]	59 participants scheduled to undergo chemotherapy consisting of anthracyclines Resistance training (RT) group = 30 Aerobic training (AT) group = 27	2x weekly, 16 weeks RT = 2–3 sets of 8–12 reps at 80% of 1-rep-max AT = 20 min of moderate-intensity continuous aerobic exercise	RT and AT significantly attenuated the fall in predicted VO2peak seen in control groups
*Ma, 2018* [65]	70 participants (intervention group = 31) undergoing anthracycline-containing therapy	Aerobic exercise 3x weekly for 16 weeks	Significant attenuation of LVEF decline in exercise group relative to control group
Foulkes et al., 2022 [54]	104 participants (intervention group = 52) 60 mg/m^2^ doxorubicin combined with 600 mg/m^2^ cyclophosphamide for 4 cycles (3 months)	3-4x weekly for 12 months Broken into 3 phases with decreasing levels of supervision Progressive loading/de-loading of volume in line with chemotherapy cycles Sessions consisted of varying combinations of **moderate-intensity endurance training** (30-60 min at heart rate [HR] 5–30 b/min below ventilatory threshold) ), **tempo training** (35 min at HR within 10 b/min of VT), **high intensity interval training** (4 × 2–4 min at > 85% maximal HR), and **moderate-to-high intensity resistance training** (2 sets x 8–15 repetitions at load equivalent to 60–85% of 1-repetition max)	Exercise associated with a net + 3.5 mL/kg/min improvement in VO2 peak Significantly greater improvements in cardiac reserve measures (change in CO, SV, LVEF and RVEF from rest to peak exercise)

Perhaps less encouragingly, however, the only other trastuzumab-specific single-arm intervention study actually reported a non-significant decrease in LVEF in exercise-treated groups, alongside worsening resting end-diastolic volume as a secondary outcome [55]. Exercise intervention in this study occurred during the first 4 months of treatment and involved aerobic exercise. However, while Hojan et al. is an RCT, Haykowsky et al. do not have a control group and thus comparison to non-intervention is lacking. Since both trials were performed during treatment it is also impossible to judge with existing data whether exercise as a pre-hab or rehab intervention may be more beneficial. Haykowsky et al. are keen to acknowledge, however, that low patient compliance in exercise populations may have diluted the true effect of intervention, reporting adherence rates as low as 59%. This is likely to be a repeating limitation of exercise as an intervention, particularly in BC populations undergoing treatment, as debilitating fatigue and weakness which increase the difficulty of training are both common symptoms of cancer and side-effects of chemotherapy [56–58]. A clinical trial exploring adherence rates of cancer patients to an exercise programme during concurrent curative cancer treatment reported dropout rates of ~ 30% and adherence rates of 47–51% [59]. In addition, a meta-analysis of RCTs examining the successes of exercise interventions in doxorubicin and trastuzumab-receiving patients highlighted that only programmes requiring ≥ 36 sessions effectively prevented LVEF decline [60]. This finding acknowledges that intervention duration may be a critical determinant of treatment success. Indeed a systematic review of reports on exercise training and LV remodelling in HF patients (with reduced ejection fraction) found that only treatments lasting ≥ 6 months significantly improved LVEF, while shorter durations conferred only modest improvements [61].

## Conclusion

The mechanisms of trastuzumab-mediated cardiotoxicity are far from simple. Despite growing interest in the field, there is still need for a better understanding of precisely how cardiomyocytes are impacted and in what ways this translates to inadequate cardiac function. Current pre-clinical findings do support exercise as an intervention which could improve metabolic capacity and contractile function of treatment-weakened hearts. Therefore, future research should focus on trastuzumab-specific animal exercise models to confirm these indications. Increased use of patient studies, including human iPSC-CMs and cardiac organoids, could help clarify for example the somewhat contradictory pathways of HER2 phosphorylation versus inhibition observed in human and animal models respectively, or to characterise potential ultrastructural changes which might be present in treated human cells.

There is currently insufficient patient-based evidence to conclude that exercise improves cardiovascular outcomes in cases of trastuzumab-mediated cardiotoxicity. Further clinical trials assessing exercise as a treatment are thus required to ascertain if this may ameliorate or prevent trastuzumab-mediated cardiotoxicity. Although a current RCT examining the effects of a 3-month exercise programme on LVEF decline in patients receiving adjuvant trastuzumab [66] will hopefully provide additional evidence for this discussion, further studies trialling longer exercise programs are required. Following insights from studies in related conditions, such as anthracycline-based cardiotoxicity and HF of other origin, it is clear that intervention duration and adherence are both critical determinants of success which must be carefully considered for future studies. More personalised approaches to exercise intervention may improve both efficacy and tolerability in the context of cardio-oncology [67], and could therefore address the current issues with adherence faced in many trials.

## Data Availability

Data sharing is not applicable to this article as no datasets were generated or analysed during the current study.
